# Pandemic (H1N1) 2009 Surveillance and Prevalence of Seasonal Influenza, Singapore

**DOI:** 10.3201/eid1601.091164

**Published:** 2010-01

**Authors:** Yee-Sin Leo, David C Lye, Timothy Barkham, Prabha Krishnan, Eillyne Seow, Angela Chow

**Affiliations:** Tan Tock Seng Hospital, Singapore

**Keywords:** Pandemic (H1N1) 2009, seasonal influenza, influenza, surveillance, viruses, Singapore, expedited, dispatch

## Abstract

On April 25, 2009, Singapore implemented strict containment measures for pandemic (H1N1) 2009 with enhanced surveillance and hospital isolation. In the first month, seasonal influenza, predominantly virus subtype H3N2, was diagnosed for 32% of patients with acute febrile respiratory illness. Our findings underscore the high prevalence of seasonal influenza in Singapore.

Tropical countries experience influenza year round, with 2 peaks corresponding to the rainy seasons (*1*). Despite this year-round activity, seasonal influenza is often neglected in tropical countries in terms of clinical diagnosis, treatment, and vaccination (*2*). In Singapore, influenza activity usually peaks in June and December (*3*). The annual all-cause death rate from seasonal influenza in Singapore has been estimated at 14.8/100,000 person-years; the proportion of deaths among persons ≥65 years of age is 11.3× higher than that among the general population (*4*). In addition, previous pandemic influenza–related excess deaths in Singapore are comparable to those in temperate countries (*5*).

In April 2009, a novel influenza A virus (H1N1) of swine origin emerged in the United States (*6*) and triggered alarm about its pandemic potential (*7*). On June 11, 2009, the World Health Organization announced that the virus had become pandemic; it is now referred to as pandemic (H1N1) 2009 virus (*8*). We report on enhanced influenza surveillance in Singapore that was implemented before the first case of pandemic (H1N1) 2009 was detected on May 26, 2009, and we describe the transition in Singapore from influenza cases caused predominantly by seasonal influenza to cases caused exclusively by pandemic (H1N1) 2009 virus.

## The Study

This surveillance study was approved by the Chairman of Medical Board, Tan Tock Seng Hospital (TTSH), Singapore. The clinical study was approved by the Domain Specific Review Board, National Healthcare Group, Singapore.

On April 25, 2009, Singapore’s Ministry of Health initiated containment measures in response to pandemic (H1N1) 2009. Travelers returning from affected countries to Singapore with acute febrile respiratory illness were screened at the TTSH emergency department, the designated screening center for pandemic (H1N1) 2009. Thermal screening was conducted at all border entry points, and the mass media publicized nationwide that persons with a risk for pandemic (H1N1) 2009 virus infection based on travel history, fever, and respiratory symptoms should go to the TTSH emergency department for screening. Combined nasal and throat swab specimens were tested with an in-house, gel-based PCR for influenza A, H1, H3, H5, N1, and N2. All specimens with subtype H1N1–positive test results underwent in-house, probe-based influenza (H1N1) 2009 PCR, and partial sequencing of the matrix gene was conducted to confirm positive PCR results.

From April 27 through May 24, 2009, a total of 300 persons (244 travelers returning from affected countries with a respiratory illness and 56 symptomatic contacts) were screened for influenza infection: 24.0% had subtype H3N2, 1.6% had seasonal subtype H1N1, and 2.7% had influenza B. The median age of patients was 36 years (range 10–70 years), and 14.7% had other illnesses (asthma [52.3% of those with other illnesses], diabetes mellitus [29.5%], heart disease [11.4%], cancer [4.5%], and HIV [4.5%]). Fever was reported for 92.9% of all screened persons, cough for 82.4%, sore throat for 57.6%, rhinorrhea for 62.4%, myalgia for 25.9%, headache for 16.5%, and gastrointestinal symptoms for 9.4%.

Two thirds of the patients with influenza-positive PCR results met the criteria for influenza-like illness (ILI) as defined by the US Centers for Disease Control and Prevention (CDC) (*9*). Patients who met these criteria were more likely than those who did not to have influenza-positive PCR results (odds ratio [OR] 8.1, 95% confidence interval [CI] 4.6–14.3, p = 0.0001). Compared with contacts who had no recent travel, travelers returning from North America were less likely to have influenza-positive PCR test results (OR 0.13, 95% CI 0.06–0.27, p = 0.0001).

To enhance detection of pandemic (H1N1) 2009, we obtained nasal and throat swab samples on May 2 and 3, 2009, from all hospitalized patients with clinically suspected pneumonia, regardless of their travel history. The samples were tested by PCR for influenza. A total of 146 patients were screened, of whom 21 (14.4%) were positive for influenza; 10.3% of the 146 patients had H3N2, 1.4% had seasonal H1N1, and 2.7% had influenza B. The median age of these patients was 67 years (range 20–95 years), and their median hospital stay at screening was 1 day (range 0–17 days). Of the patients, 52% were male, and 86% had other illnesses. At the time of admission, 90% of patients had a fever, 76% had a cough, 24% had a sore throat, 52% had rhinorrhea, 24% had myalgia, and 19% had headache. Findings on chest radiographs were abnormal for 10 patients (48%), of whom 5 had findings consistent with pneumonia. All but 1 patient were treated with antimicrobial drugs; none was given antiviral drug therapy. The median hospital stay was 5 days (range 1–38 days). Two patients died; both had multiple illnesses.

In addition, during May 2–8, 2009, as part of nationwide enhanced influenza surveillance, nasal and throat swab specimens from patients screened at the TTSH emergency department were tested by PCR for influenza virus. The patients had fever or respiratory symptoms but no history of travel to an affected country. Overall, 95 patients were screened, of whom 30 (31.6%) had positive results for influenza; 24.2% of the 95 patients had H3N2 and 7.4% had influenza B. Fever was reported for 69.5% of the 95 patients, cough for 75.8%, sore throat for 56.8%, rhinorrhea for 58.9%, myalgia for 16.8%, and headache for 44.2%. Of the 30 patients with PCR results positive for influenza, 22 (73%) met the criteria for ILI. Compared with patients who did not meet these criteria, patients who did meet them were 3× more likely to have influenza-positive PCR results (OR 3.02, 95% CI 1.17–7.75, p = 0.019). Only 6 (6.3%) of the 95 patients had self-reported influenza vaccination in the preceding 6 months.

Screening of patients with ILI symptoms continued at the TTSH emergency department, and from May 3 through June 13, 2009, only seasonal influenza (predominantly virus subtype H3N2) was detected. Pandemic (H1N1) 2009 was first detected during the week beginning June 14, and the weekly incidence rapidly increased until the week ending July 25, when all influenza cases were caused by pandemic (H1N1) 2009 virus (Figure).

**Figure:**
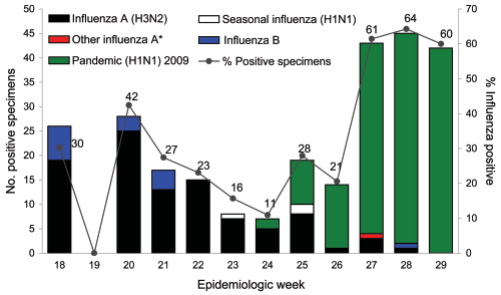
Influenza-positive test results for surveillance samples obtained at the emergency department, Tan Tock Seng Hospital, Singapore, May 3–July 25, 2009. An epidemiologic week starts on a Sunday and ends on a Saturday (e.g., week 18 started on May 3 and week 29 on July 19). *Undetermined influenza A subtypes.

By July 25, 2009, a total of 838 patients with pandemic (H1N1) 2009 virus infection had been seen at the TTSH emergency department. The median age of patients was 22 years (range 10–90 years). Fever was reported for 85.3%, cough for 87.2%, sore throat for 55.4%, rhinorrhea for 41.6%, myalgia for 11.1%, and headache for 11.0%; 57% of patients met the CDC criteria for ILI. Patients with pandemic (H1N1) 2009 were significantly younger (p = 0.0001) than patients with seasonal influenza, but the proportion with ILI in each group was similar (OR 0.65, 95% CI 0.41–1.04, p = 0.071).

## Conclusions

Each year in the United States, seasonal influenza accounts for >200,000 hospitalizations and 41,000 deaths, and it is the seventh leading cause of death (*10*). The effect of seasonal influenza in the tropics is less well studied. In Thailand, influenza was detected in 11%–12.5% of patients with community-acquired pneumonia and in 23% of outpatients with ILI (*11*,*12*).

Enhanced influenza surveillance in Singapore in response to pandemic (H1N1) 2009 yielded a high prevalence of seasonal influenza: 28.3% (85 of 300) among returning travelers and their contacts with respiratory symptoms, 14.4% (21 of 146) among hospitalized patients with suspected pneumonia, and 31.6% (30 of 95) among patients self-reporting to the TTSH emergency department with fever and respiratory symptoms but without a history of travel to affected countries. These findings reflect the peak local influenza season (*2*,*4*,*5*). The risk of having seasonal influenza was lower among travelers returning from North America, a finding that was consistent with the timing of the peak local influenza season and the noninfluenza season in the Northern Hemisphere.

Of 21 patients with influenza-positive PCR results, only 5 had radiographic evidence of pneumonia despite having a pneumonia diagnosis based on an acute history of fever and cough. No patients, including those with positive test results, were treated with antiviral drugs. This finding, in addition to low influenza vaccine coverage in Singapore, reflects the underappreciation of influenza by doctors in this country.

Of note, 67%–73% of the patients with influenza-positive PCR results met the CDC ILI criteria during the study period; this finding may guide testing for seasonal influenza during peak influenza seasons in May–June and December. Its value in nonpeak seasons, however, requires further evaluation because influenza occurs year-round in the tropics (*1*). Temperature >38°C and either cough or sore throat (*13*) were the most specific screening criteria and had the best positive predictive value; temperature of 37.5°C and either cough, sore throat, or rhinorrhea were the most sensitive screening criteria and had the best negative predictive value for influenza (Table).

**Table:** Performance of influenza screening criteria for travelers returning to Singapore and their contacts, April 27­–May 24, 2009

Screening criteria	Sensitivity, %	Specificity, %	Positive predictive value, %	Negative predictive value, %
Temperature >37.5°C and cough or sore throat	74.1	71.6	50.8	87.5
Temperature >37.5°C and cough, sore throat, or rhinorrhea	81.2	71.2	52.7	90.5
Temperature >37.8°C and cough or sore throat (*9*)	67.1	80.0	57.0	86.0
Temperature >38°C and cough or sore throat (*13*)	54.1	87.0	62.2	82.7

Our findings highlight a high prevalence of seasonal influenza during peak times in Singapore; the prevalence is comparable to that during typical influenza seasons in temperate countries in the Southern Hemisphere. This prevalence underscores the need for not neglecting seasonal influenza and for a more robust surveillance system to be in place for community and hospital monitoring in Singapore.
